# Benzene-1,3,5-triol at 105 K

**DOI:** 10.1107/S1600536808030638

**Published:** 2008-09-27

**Authors:** Carl Henrik Görbitz, Massoud Kaboli, Matthew Lovell Read, Kristian Vestli

**Affiliations:** a Department of Chemistry, University of Oslo, PO Box 1033 Blindern, N-0315 Oslo, Norway

## Abstract

The structure of the title compound, C_6_H_6_O_3_, has been redetermined at low temperature [room-temperature structure: Maartmann-Moe (1965 ▶). *Acta Cryst*. **19**, 155–157]. The mol­ecule is planar with approximate *D*_3*h*_ point symmetry, yet it crystallizes in the chiral ortho­rhom­bic space group *P*2_1_2_1_2_1_ with a three-dimensional hydrogen-bonding network containing infinite O—H⋯O—H⋯O—H chains.

## Related literature

For the structure at room temperature, see: Maartmann-Moe (1965 ▶). For the hydrate structure, see: Wallwork & Powell (1957 ▶).
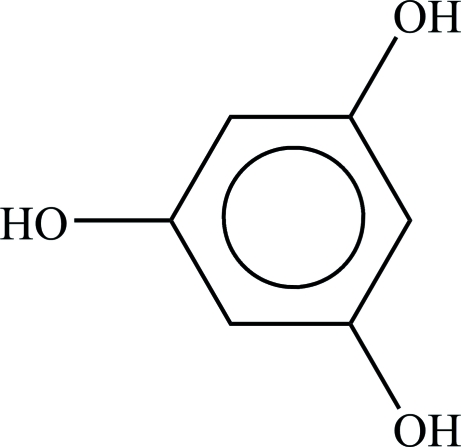


## Experimental

### 

#### Crystal data

C_6_H_6_O_3_
*M* *_r_* = 126.11
Orthorhombic, 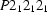

*a* = 4.7778 (2) Å
*b* = 9.3581 (4) Å
*c* = 12.4433 (6) Å
*V* = 556.35 (4) Å^3^
*Z* = 4
Mo *K*α radiation
μ = 0.12 mm^−1^
*T* = 105 (2) K
0.20 × 0.08 × 0.05 mm

#### Data collection

Siemens SMART CCD diffractometer
Absorption correction: multi-scan (*SADABS*; Sheldrick, 1996 ▶) *T*_min_ = 0.916, *T*_max_ = 0.997
6178 measured reflections
743 independent reflections
728 reflections with *I* > 2σ(*I*)
*R*_int_ = 0.014

#### Refinement

*R*[*F* ^2^ > 2σ(*F* ^2^)] = 0.026
*wR*(*F* ^2^) = 0.081
*S* = 1.13
743 reflections
91 parameters
H atoms treated by a mixture of independent and constrained refinement
Δρ_max_ = 0.27 e Å^−3^
Δρ_min_ = −0.20 e Å^−3^

### 

Data collection: *SMART* (Bruker, 1998 ▶); cell refinement: *SAINT-Plus* (Bruker, 2001 ▶); data reduction: *SAINT-Plus*; program(s) used to solve structure: *SHELXTL* (Sheldrick, 2008 ▶); program(s) used to refine structure: *SHELXTL*; molecular graphics: *SHELXTL*; software used to prepare material for publication: *SHELXTL*.

## Supplementary Material

Crystal structure: contains datablocks I, global. DOI: 10.1107/S1600536808030638/bi2305sup1.cif

Structure factors: contains datablocks I. DOI: 10.1107/S1600536808030638/bi2305Isup2.hkl

Additional supplementary materials:  crystallographic information; 3D view; checkCIF report

**Fig. 1.**
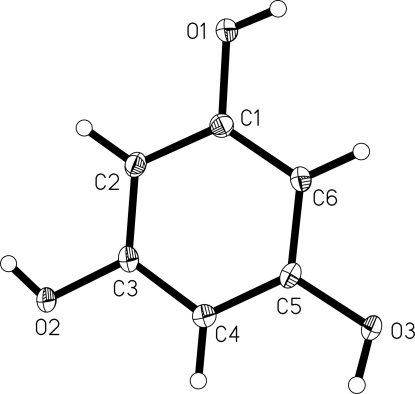
The molecular structure of the title compound. Displacement ellipsoids are shown at the 50% probability level and H-atoms are shown as spheres of arbitrary size.

Enhanced figure: interactive version of Fig. 1

## Figures and Tables

**Table 1:** Hydrogen-bond geometry (Å, °)

*D*—H⋯*A*	*D*—H	H⋯*A*	*D*⋯*A*	*D*—H⋯*A*
O1—H1⋯O3^i^	0.83 (2)	1.94 (2)	2.7426 (13)	164 (2)
O2—H2⋯O1^ii^	0.79 (2)	1.97 (2)	2.7424 (14)	169 (2)
O3—H3⋯O2^iii^	0.86 (2)	1.85 (2)	2.7086 (16)	173.3 (17)

Symmetry codes: (i) 
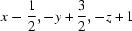
; (ii) 
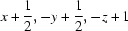
; (iii) 
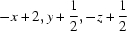
.

**Fig. 2.**
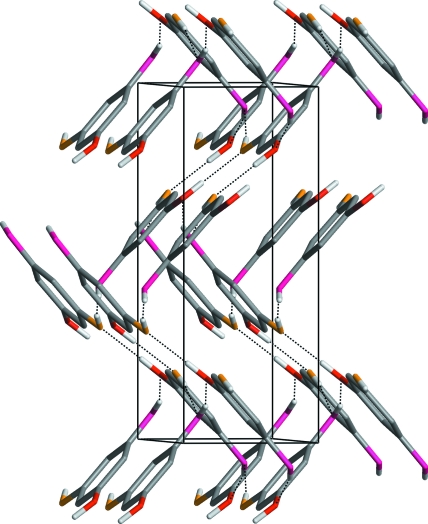
Molecular packing and unit cell of the title compound viewed along the b axis. Hydrogen bonding is indicated by dashed lines, H-atoms bonded to C have been omitted for clarity. The three different hydroxylic O atoms have been depicted in different colours.

**Fig. 3.**
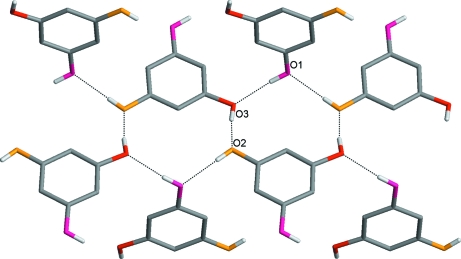
Detail of the hydrogen bonding pattern showing infinite hydrogen-bonded chains.

## supplementary crystallographic information

### Comment

The structure of the benzene-1,3,5-triol, commonly known as phloroglucinol, is shown in Fig. 1. The molecule is essentially planar, with *D*_3_*_h_* point symmetry, having only small out-of-plane rotations for the hydroxyl groups. Rather than forming a layer-like structure, a folded molecular aggregation pattern is observed in the crystal (Fig. 2) giving a three-dimensional hydrogen-bonding pattern. The three hydrogen bonds listed in Table 1 form an infinite zigzag chain along the *b* axis as shown in Fig. 3. The agreement with the original structure determination (Maartmann-Moe, 1965) is generally good, but with some significant changes in the hydrogen bonding geometries.

Benzene-1,3,5-triol has also been crystallized as a dihydrate, which is divided into layers with water molecules as connectors (Wallwork & Powell, 1957).

### Experimental

The title compound was obtained from Fluka. Crystals were grown by diffusion of hexane into 30 µl of a solution containing 2.1 mg benzene-1,3,5-triol and 1.3 mg triazin in 3-methyl-2-butanone.

### Refinement

Positional parameters were refined for hydroxylic H atoms, while H atoms bonded to C were positioned with idealized geometry and C—H distance 0.95 Å. *U*_iso_ values were 1.5*U*_eq_(O) and 1.2*U*_eq_(C). In the absence of significant anomalous scattering effects, 1585 Friedel pairs were merged.

### Crystal data

**Table:**

C_6_H_6_O_3_	*F*(000) = 264
*M**_r_* = 126.11	*D*_x_ = 1.506 Mg m^−^^3^
Orthorhombic, *P*2_1_2_1_2_1_	Mo *K*α radiation, λ = 0.71073 Å
Hall symbol: P 2ac 2ab	Cell parameters from 4652 reflections
*a* = 4.7778 (2) Å	θ = 2.7–27.1°
*b* = 9.3581 (4) Å	µ = 0.12 mm^−^^1^
*c* = 12.4433 (6) Å	*T* = 105 K
*V* = 556.35 (4) Å^3^	Needle, colourless
*Z* = 4	0.20 × 0.08 × 0.05 mm

### Data collection

**Table:**

Siemens SMART CCD diffractometer	743 independent reflections
Radiation source: fine-focus sealed tube	728 reflections with *I* > 2σ(*I*)
graphite	*R*_int_ = 0.014
ω scans	θ_max_ = 27.1°, θ_min_ = 2.7°
Absorption correction: multi-scan (SADABS; Sheldrick, 1996)	*h* = −6→6
*T*_min_ = 0.916, *T*_max_ = 0.997	*k* = −11→11
6178 measured reflections	*l* = −15→15

### Refinement

**Table:**

Refinement on *F*^2^	Primary atom site location: structure-invariant direct methods
Least-squares matrix: full	Secondary atom site location: difference Fourier map
*R*[*F*^2^ > 2σ(*F*^2^)] = 0.027	Hydrogen site location: inferred from neighbouring sites
*wR*(*F*^2^) = 0.081	H atoms treated by a mixture of independent and constrained refinement
*S* = 1.13	*w* = 1/[σ^2^(*F*_o_^2^) + (0.054*P*)^2^ + 0.1017*P*] where *P* = (*F*_o_^2^ + 2*F*_c_^2^)/3
743 reflections	(Δ/σ)_max_ < 0.001
91 parameters	Δρ_max_ = 0.27 e Å^−^^3^
0 restraints	Δρ_min_ = −0.20 e Å^−^^3^

### Special details

**Table:**

Geometry. All e.s.d.'s (except the e.s.d. in the dihedral angle between two l.s. planes) are estimated using the full covariance matrix. The cell e.s.d.'s are taken into account individually in the estimation of e.s.d.'s in distances, angles and torsion angles; correlations between e.s.d.'s in cell parameters are only used when they are defined by crystal symmetry. An approximate (isotropic) treatment of cell e.s.d.'s is used for estimating e.s.d.'s involving l.s. planes.
Refinement. Data were collected by measuring three sets of exposures with the detector set at 2θ = 29°, crystal-to-detector distance 5.00 cm. Refinement of *F*^2^ against ALL reflections.

### Fractional atomic coordinates and isotropic or equivalent isotropic displacement parameters (Å^2^)

**Table:**

	*x*	*y*	*z*	*U*_iso_*/*U*_eq_	
O1	0.2140 (2)	0.48267 (10)	0.56722 (8)	0.0160 (3)	
H1	0.189 (5)	0.560 (2)	0.5979 (15)	0.024*	
O2	0.8083 (3)	0.25657 (10)	0.31075 (8)	0.0167 (3)	
H2	0.760 (5)	0.189 (2)	0.3432 (14)	0.025*	
O3	0.7415 (2)	0.76483 (10)	0.32139 (8)	0.0160 (3)	
H3	0.884 (5)	0.7549 (19)	0.2792 (16)	0.024*	
C1	0.3967 (3)	0.49437 (14)	0.48167 (11)	0.0135 (3)	
C2	0.5004 (3)	0.36808 (13)	0.43819 (11)	0.0141 (3)	
H21	0.4423	0.2779	0.4652	0.017*	
C3	0.6913 (3)	0.37730 (13)	0.35413 (10)	0.0135 (3)	
C4	0.7758 (3)	0.50800 (15)	0.31202 (11)	0.0150 (3)	
H41	0.9054	0.5126	0.2542	0.018*	
C5	0.6651 (3)	0.63152 (13)	0.35689 (10)	0.0134 (3)	
C6	0.4732 (3)	0.62700 (13)	0.44129 (11)	0.0137 (3)	
H61	0.3970	0.7124	0.4704	0.016*	

### Atomic displacement parameters (Å^2^)

**Table:**

	*U*^11^	*U*^22^	*U*^33^	*U*^12^	*U*^13^	*U*^23^
O1	0.0199 (5)	0.0107 (5)	0.0173 (5)	−0.0003 (4)	0.0057 (4)	−0.0006 (3)
O2	0.0217 (5)	0.0090 (5)	0.0194 (5)	0.0017 (4)	0.0058 (4)	0.0000 (4)
O3	0.0209 (6)	0.0093 (5)	0.0177 (5)	−0.0015 (4)	0.0054 (5)	0.0009 (3)
C1	0.0127 (6)	0.0142 (6)	0.0136 (6)	−0.0006 (6)	−0.0006 (5)	−0.0004 (5)
C2	0.0152 (6)	0.0109 (6)	0.0161 (6)	−0.0009 (5)	−0.0003 (6)	0.0017 (5)
C3	0.0142 (6)	0.0108 (6)	0.0154 (6)	0.0014 (6)	−0.0013 (6)	−0.0012 (5)
C4	0.0158 (6)	0.0141 (6)	0.0150 (6)	0.0000 (5)	0.0032 (5)	0.0003 (5)
C5	0.0152 (7)	0.0104 (6)	0.0145 (6)	−0.0017 (6)	−0.0014 (6)	0.0012 (5)
C6	0.0151 (6)	0.0110 (6)	0.0152 (6)	0.0005 (5)	0.0008 (6)	−0.0020 (5)

### Geometric parameters (Å, °)

**Table:**

O1—C1	1.3808 (17)	C2—C3	1.3905 (19)
O1—H1	0.83 (2)	C2—H21	0.9500
O2—C3	1.3712 (16)	C3—C4	1.3905 (18)
O2—H2	0.79 (2)	C4—C5	1.3884 (19)
O3—C5	1.3730 (15)	C4—H41	0.9500
O3—H3	0.86 (2)	C5—C6	1.3945 (19)
C1—C6	1.3881 (17)	C6—H61	0.9500
C1—C2	1.3910 (18)		
			
C1—O1—H1	112.2 (14)	C2—C3—C4	121.90 (12)
C3—O2—H2	109.9 (14)	C5—C4—C3	118.05 (12)
C5—O3—H3	107.9 (12)	C5—C4—H41	121.0
O1—C1—C6	121.10 (11)	C3—C4—H41	121.0
O1—C1—C2	117.24 (11)	O3—C5—C4	121.72 (12)
C6—C1—C2	121.67 (12)	O3—C5—C6	116.41 (11)
C3—C2—C1	118.27 (12)	C4—C5—C6	121.87 (12)
C3—C2—H21	120.9	C1—C6—C5	118.22 (12)
C1—C2—H21	120.9	C1—C6—H61	120.9
O2—C3—C2	120.83 (12)	C5—C6—H61	120.9
O2—C3—C4	117.26 (12)		
			
O1—C1—C2—C3	−178.06 (12)	O1—C1—C6—C5	178.09 (12)
C6—C1—C2—C3	1.8 (2)	C2—C1—C6—C5	−1.8 (2)
C1—C2—C3—O2	177.59 (12)	O3—C5—C6—C1	−178.14 (12)
C1—C2—C3—C4	−1.1 (2)	C4—C5—C6—C1	1.0 (2)
O2—C3—C4—C5	−178.32 (13)	H1—O1—C1—C6	−13.0 (16)
C2—C3—C4—C5	0.4 (2)	H2—O2—C3—C2	−4.3 (16)
C3—C4—C5—O3	178.75 (13)	H3—O3—C5—C4	−10.8 (14)
C3—C4—C5—C6	−0.4 (2)		

### Hydrogen-bond geometry (Å, °)

**Table:**

*D*—H···*A*	*D*—H	H···*A*	*D*···*A*	*D*—H···*A*
O1—H1···O3^i^	0.83 (2)	1.94 (2)	2.7426 (13)	164 (2)
O2—H2···O1^ii^	0.79 (2)	1.97 (2)	2.7424 (14)	169 (2)
O3—H3···O2^iii^	0.86 (2)	1.85 (2)	2.7086 (16)	173.3 (17)

Symmetry codes: (i) *x*−1/2, −*y*+3/2, −*z*+1; (ii) *x*+1/2, −*y*+1/2, −*z*+1; (iii) −*x*+2, *y*+1/2, −*z*+1/2.
